# New perspectives on the potential of tetrandrine in the treatment of non-small cell lung cancer: bioinformatics, Mendelian randomization study and experimental investigation

**DOI:** 10.18632/aging.205384

**Published:** 2024-01-04

**Authors:** Jihang Luo, Xiaocong Mo, Di Hu, Yin Li, Meng Xu

**Affiliations:** 1 Department of Oncology, The First Affiliated Hospital of Jinan University, Jinan University, Guangzhou, China; 2 Department of Infectious Diseases, Affiliated Hospital of Zunyi Medical University, Zunyi, China; 3 Department of Neurology and Stroke Centre, The First Affiliated Hospital of Jinan University, Guangzhou, China

**Keywords:** tetrandrine, non-small cell lung cancer (NSCLC), bioinformatics, summary-data-based Mendelian randomization (SMR), CCNA2

## Abstract

Background: Although there are numerous treatment methods for NSCLC, long-term survival remains a challenge for patients. The objective of this study is to investigate the role and causal relationship between the target of tetrandrine and non-small cell lung cancer (NSCLC) through transcriptome and single-cell sequencing data, summary-data-based Mendelian Randomization (SMR) and basic experiments. The aim is to provide a new perspective for the treatment of NSCLC.

Methods: We obtained the drug target gene of tetrandrine through the drug database, and then used the GSE19188 data set to obtain the NSCLC pathogenic gene, established a drug-disease gene interaction network, screened out the hub drug-disease gene, and performed bioinformatics and tumor cell immune infiltration analysis. Single-cell sequencing data (GSE148071) to determine gene location, SMR to clarify causality and drug experiment verification.

Results: 10 drug-disease genes were obtained from 213 drug targets and 529 disease genes. DO/GO/KEGG analysis showed that the above genes were all related to the progression and invasion of NSCLC. Four drug-disease genes were identified from a drug-disease PPI network. These four genes were highly expressed in tumors and positively correlated with plasma cells, T cells, and macrophages. Subsequent single-cell sequencing data confirmed that these four genes were distributed in epithelial cells, and SMR analysis revealed the causal relationship between CCNA2 and CCNB1 and the development of NSCLC. The final molecular docking and drug experiments showed that CCNA2 and CCNB1 are key targets for tetrandrine in the treatment of NSCLC.

## INTRODUCTION

According to the 2018-2020 global cancer statistics [1, 2], Among all malignant tumors, lung cancer has one of the highest incidence rates (over 10%) and the highest mortality rate (over 18.4%). Non-small cell lung cancer (NSCLC) is the predominant pathological type of lung cancer, which includes adenocarcinoma, squamous cell carcinoma, and large cell carcinoma [3, 4]. Surgery, radiation therapy, chemotherapy, targeted therapy, and immunotherapy are among the primary treatment options for NSCLC. Early NSCLC can usually be treated with surgery and stereotactic radiotherapy, but clinically most patients are diagnosed at an advanced stage and have lost the chance of radical cure. In recent years, targeted therapy has made important progress in non-squamous non-small cell lung cancer, especially the efficacy of targeted drugs targeting mutated genes such as EGFR, ALK, ROS1, and BRAF has been fully confirmed [5]. At the same time, with the widespread application of immune checkpoint inhibitors in the treatment of NSCLC, the overall survival of a considerable number of patients has been prolonged [6]. However, for most NSCLC patients, drug resistance is an unavoidable problem with the above therapies, so researchers need to find other treatment strategies.

Traditional Chinese medicine has a long history in the treatment of non-small cell lung cancer, and has accumulated rich clinical experience. CATLA study showed that the Chinese herbal medicine Yiqi Yangyin Jiedu Decoction (YYJD) can delay the occurrence of acquired resistance to the first-generation EGFR-TKI, which provides a new idea for the treatment of non-small cell lung cancer [7]. Studies have found that theabrownin (TB) is a pigment active substance in green tea, which has the ability to phosphorylate and activate MAPK/JNK pathway-related proteins, and at the same time inhibit epithelial-mesenchymal transition-related genes and pro-apoptotic molecules, p53 signaling pathway and MAPK/JNK signaling pathway, thereby effectively inhibiting the growth of NSCLC cells in xenograft models [8]. Cinobufacin (CNB) is a cardiotonic steroid or bufonilide extracted from Bufonis, which has detoxification, swelling, and analgesic effects. Studies have found that cinobufacin induces FOXO1 by inhibiting non-small cell lung cancer A549 cells G9a Regulates apoptosis, proliferation, migration and invasion [9].

Stephania tetrandra S. Moore is a traditional Chinese medicinal material. Its root is used as medicine to reduce edema and relieve pain. It can be used for dysuria and hypertension [10]. Tetrandrine is indicated for rheumatic pain, arthralgia, neuralgia, silicosis, and it can also be used in small doses in lung cancer radiotherapy [11]. Through the action of tetrandrine, the acidic environment of lysosomes is neutralized, thereby affecting the degradation process of the autophagy pathway, and at the same time inducing the apoptosis of prostate cancer, liver cancer, kidney cancer and bladder cancer cells [12]. Studies have found that tetrandrine can upregulate the mRNA and protein levels of BMP9 in colon cancer cells, and finally inhibit the proliferation of colon cancer cells through BMP9/PTEN/PI3K/AKT signaling [13]. It is reported that tetrandrine can enhance the expression of PARP, Bax, ICAM-1 and VEGF, and effectively inhibit the growth and induce apoptosis of A549 lung cancer cells by activating the VEGF/HIF-1α pathway [14]. It can be seen from the above that tetrandrine has a wide range of anticancer effects.

Variations in an individual’s genome can affect drug metabolism, absorption, distribution, and excretion, thereby affecting drug efficacy and adverse effects [15]. Therefore, knowing an individual’s drug-disease genetic information can help doctors better choose drugs and drug dosages to improve treatment efficacy and reduce the risk of adverse reactions. In addition, drug-disease genetic information can also be used to develop more personalized treatment strategies and drugs. To assess the potential efficacy of tetrandrine in NSCLC, we constructed an interaction network between tetrandrine drug targets and NSCLC oncogenes. Through screening, we identified four hub genes and investigated their relevance to immune cells. Leveraging single-cell sequencing data, we elucidated the drug’s cell-type specificity and employed SMR analysis to establish the causal relationship between these genes and NSCLC. Our findings confirmed the ability of tetrandrine to inhibit proliferation of NSCLC tumor cells and identified important targets that can inform current clinical treatments for NSCLC.

## MATERIALS AND METHODS

### Drug target acquisition and functional analysis

From Comparative Toxicogenomics Database (CTD) (http://ctdbase.org/) [16], SwissTargetPrediction (http://www.swisstargetprediction.ch/) [17], BindingDB (http://bindingdb.org/bind/) [18], TargetNet (http://targetnet.scbdd.com/home/index/) [19] four databases downloaded the target information of tetrandrine. In order to standardize and harmonize information obtained from various databases, we have retained the drug gene names from these database results while eliminating extraneous expression data. We then used a Venn diagram to display the combined results of drug genes in the above database. Disease Ontology (DO)/Gene Ontology (GO)/Kyoto Encyclopedia of Genes and Genomes (KEGG) analyzes were performed using these pharmacogenes, respectively.

### NSCLC data obtain and analysis

NSCLC patient information and transcriptome data come from the GSE19188 dataset (https://www.ncbi.nlm.nih.gov/geo/) [20]. The dataset encompasses genetic data from a cohort of 91 patients afflicted with NSCLC, comprising 91 tumor specimens and associated information from 65 neighboring normal lung tissue samples. R was used to normalized the transcriptome data in this data set, and then the “limma” R package was used to screen the differential genes in the disease according to the standard of│logFC│*>1*, *P<0.05*. The “WGCNA” R package was used to cluster genes with similar expression patterns in GSE19188 to form different modules, and the relationship between genes and phenotypes or traits in the modules was analyzed. Finally, the intersection of the differential gene and the MEturquoise module genes analyzed by WGCNA was used as the disease gene. GO and KEGG enrichment analysis of disease genes to elucidate their functions in NSCLC.

### Drug-disease joint analysis

Put all the drug and disease genes through the STRING database (https://string-db.org/) [21] to create a protein-protein interaction (PPI) network with a confidence interval of 0.4, and delete independent genes that do not interact with other genes. Take the intersection of drug gene and disease gene as drug-disease gene. Then, use Cytoscape version 3.8.0 to process the PPI network, and use MCODE to set the Degree cutoff value to 2, and the rest to the default settings to get a subnetwork consisting of 124 genes. In order to further screen hub genes, a core sub-network composed of 41 genes was screened again through the STRING database and MCODE in Cytoscape according to the above criteria. DO/GO/KEGG analyzes were performed using these hub genes, respectively. At the same time, R was used to describe the expression of 41 genes in tumor and normal tissues in NSCLC. The “ pROC “ R package established receiver operating characteristic (ROC) curves to verify the performance of hub genes.

### Immune infiltration analysis

We used the CIBERSORT algorithm to analyze the level of immune cell infiltration in normal and tumor tissues in the GSE19188 dataset. In addition, we also evaluated the correlation between each hub gene and immune cells using the “IOBR” R package to determine whether these genes could be used as new biomarkers in immunotherapy. These measures help to gain insight into the immune signature of tumors and provide the basis for personalized treatment of immunotherapy.

### Drug-disease gene and single cell sequencing analysis

The single-cell sequencing data set (GSE148071) was obtained from the GEO database (https://www.ncbi.nlm.nih.gov/geo/) [22], the dataset comprises 42 tumor samples from non-small cell lung cancer patients obtained through methods such as percutaneous biopsy, bronchoscopy, or superficial lymph node biopsy, along with their associated single-cell sequencing data. Then the single-cell data were processed using R packages such as “Seurat”, “harmony”, “UCell”, “irGSEA”, “GSVA”, and “GSEABase”. Percentages of mitochondria and rRNA are calculated by the PercentageFeatureSet function and ensure expression of more than 200 genes and <10,000 genes per cell with mitochondrial content <20%. All gene expression levels were processed by the “LogNormalize” function, and then the top 2000 highly variable genes were screened out using “FindVariableFeatures” function. After normalizing all genes, Principal Component Analysis (PCA) and Uniform Manifold Approximation and Projection (UMAP) were used for data dimensionality reduction. Cell annotation via HumanPrimaryCellAtlasData. Finally, according to the standard of min.pct = 0.25, logfc.threshold = 0.25, the differentially expressed genes are retained and combined with the drug-disease gene list to display the distribution map to see which cell subsets the drug-disease gene acts on.

### Summary-data-based Mendelian randomization

This study is based on publicly available summary-level data from genome-wide association studies (GWAS) and expression quantitative trait loci (eQTL) studies. These studies have been approved by relevant institutional review boards and informed consent has been obtained from the participants. Four hub genes (CCNB1, CCNA2, BIRC5, and AURKB) were selected as exposures, and their available eQTLs were used as genetic instruments for each gene. The eQTL summary-level data were sourced from eQTLGen (https://www.eqtlgen.org/) [23]. We identified common eQTLs (minor allele frequency [MAF] >1%, *P < 5.0 × 10^-8^*) significantly associated with the expression of the four genes in blood samples. The summary-level data for non-small cell lung cancer (NSCLC) GWAS were obtained from FINNGEN (https://www.finngen.fi/en) [24] R8, with a total of 263,448 samples, including 3,865 NSCLC cases and 259,583 controls. Subsequently, SMR software version 1.31 (https://yanglab.westlake.edu.cn/software/smr/#Download) [25] was used for allele harmonization and analysis. We utilized the F-statistic to assess weak instrument variable effects and included SNPs with *F > 10* to minimize weak instrument bias. For the SMR method, a *P-value ≥0.01* from the heterogeneity in dependent instruments (HEIDI) test indicates no heterogeneity [26]. This step was performed to ensure the reliability and stability of the results.

### Validation of all cell lines

Origin and Acquisition: The A549 and H1299 cell lines were acquired on April 5, 2023 from Dr. Mo’s laboratory. Testing and Authentication: Both the A549 and H1299 cell lines have undergone rigorous testing and authentication to ensure their identity and purity. The authentication process involved the use of standard molecular biology techniques. Method of Testing: The authentication of the A549 and H1299 cell lines was conducted using DNA profiling, specifically through short tandem repeat (STR) analysis. This method allows for the comparison of the cell line’s STR profile with known reference profiles to confirm its identity. Last Testing and Verification: The A549 and H1299 cell lines were last tested and verified for authentication on May 30, 2023. This recent authentication confirms the integrity and fidelity of the cell lines used in this study. We affirm that the above information accurately reflects the origin, authentication, testing method, and recent verification status of the A549 and H1299 cell lines used in our research.

### Cytotoxicity, apoptosis assay and Edu incorporation assay

The NSCLC cell lines A549 and H1299 were maintained in 1640 medium (Hyclone, USA) containing 10% FBS (Gibco, USA) at 37° C in an atmosphere of 5% CO_2_. These cells were digested with pancreatin (Life-iLab, Shanghai, China). NSCLC cells were primarily seeded in several 96-well plates, and after tetrandrine treatment, CCK-8 reagent was added and operated according to the manufacturer’s instructions. Cell apoptosis was conducted by Flow cytometry using Annexin V– FITC Detection Kit (Solarbio, Beijing, China). EdU incorporation assay was employed to conduct the cell proliferation capacity of NSCLC cells treated with tetrandrine (Abbkine, China) according to the manufacturer’s instructions. Finally, images were obtained and preserved under the confocal microscope.

### Molecular docking

First use the punchem website (https://pubchem.ncbi.nlm.nih.gov/) [27] to obtain the 2D structure of tetrandrine, then use ChemBio3D Ultra 14.0.0.117 to convert it into a 3D structure. The protein structure of CCNA2 and CCNB1 were obtained from the RCSB database [28]. AutoDockTools 1.5.6 (https://autodock.scripps.edu/) was utilized to prepare the receptor protein and ligand molecules, including hydrogen addition, partial charge allocation, and protonation state assignment. During molecular docking, the XYZ grid coordinates of CCNA2 were set to 18.553, 62.684, and 77.232, and the XYZ grid coordinates of CCNB1 were set to -60.067, 31.797, and -14.94, with a grid length of 40Å in each direction. The binding modes of the ligand molecules were generated using Autodock Vina [29], with a exhaustiveness setting of 8, maximum allowable energy range of 5 kcal/mol, and maximum output conformations of 20. The Protein-Ligand Interaction Profiler online site [30] is used to analyze non-covalent interactions between molecules and their ligands. PyMOL was used to process the resulting plots.

## RESULTS

### Functional enrichment analysis of drug targets

**Figure 1:**
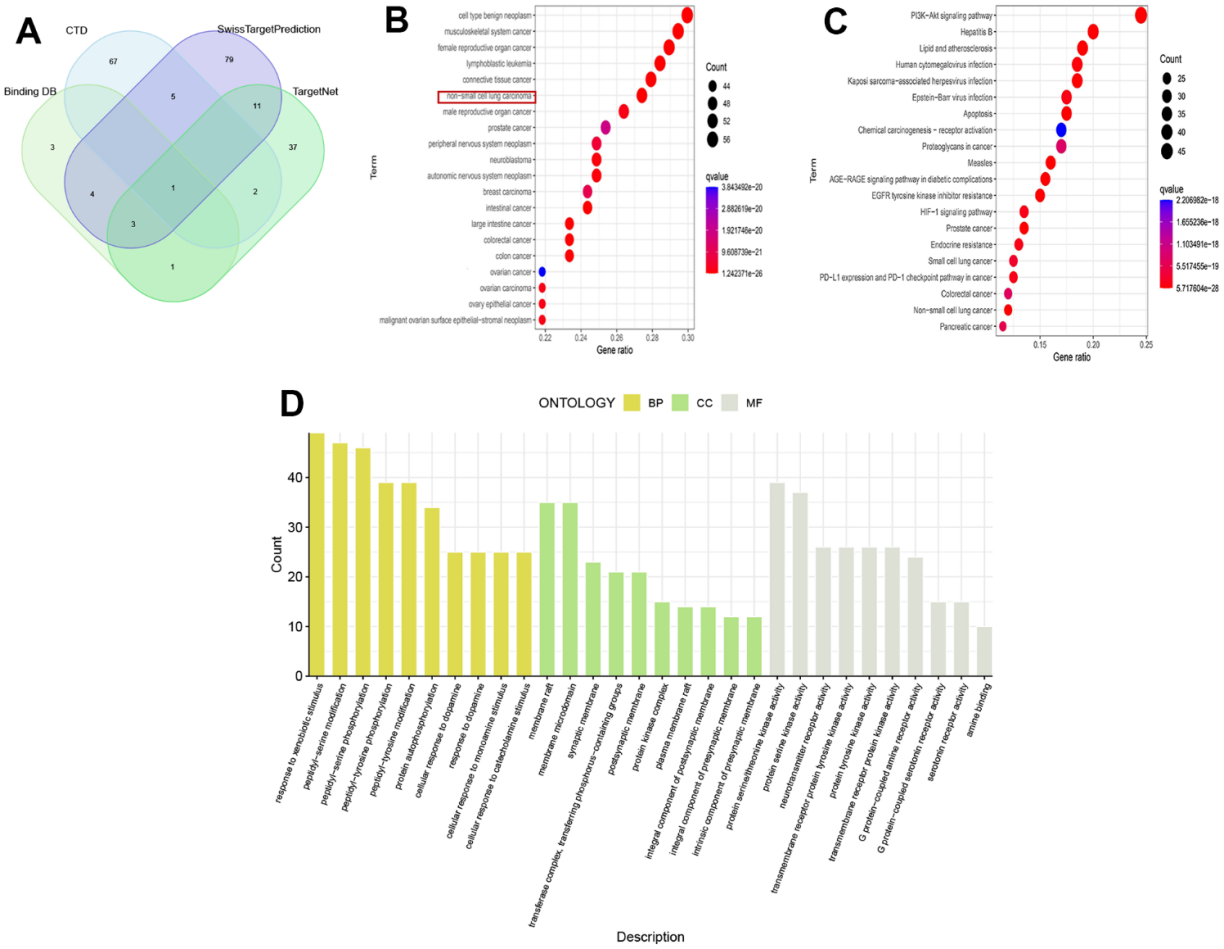
**Analysis of drug targets.** (**A**) Venn diagram of drug targets; (**B**) DO analysis; (**C**) KEGG analysis; (**D**) GO analysis.

213 drug target genes were identified from four drug target databases, including CTD, SwissTargetPrediction, Binding DB, and TargetNet, and their intersection was shown in Figure 1A. The results of DO analysis indicated that these drug genes mainly functioned in various cancers, such as non-small cell lung cancer, connective tissue cancer, and musculoskeletal system cancer (Figure 1B). KEGG analysis revealed that these genes were enriched in tumor pathways closely related to tumor pathways, including EGFR tyrosine kinase inhibitor resistance, PI3K-Akt signaling pathway, PD-L1 expression, and PD-1 checkpoint pathway (Figure 1C). Furthermore, GO analysis showed that drug genes were mainly enriched in biological processes of peptide-serine/threonine modification (BP), protein kinase complex in cellular components (CC), and protein serine/threonine kinase activity in molecular function (MF) (Figure 1D).

### Acquisition and analysis of disease genes

**Figure 2:**
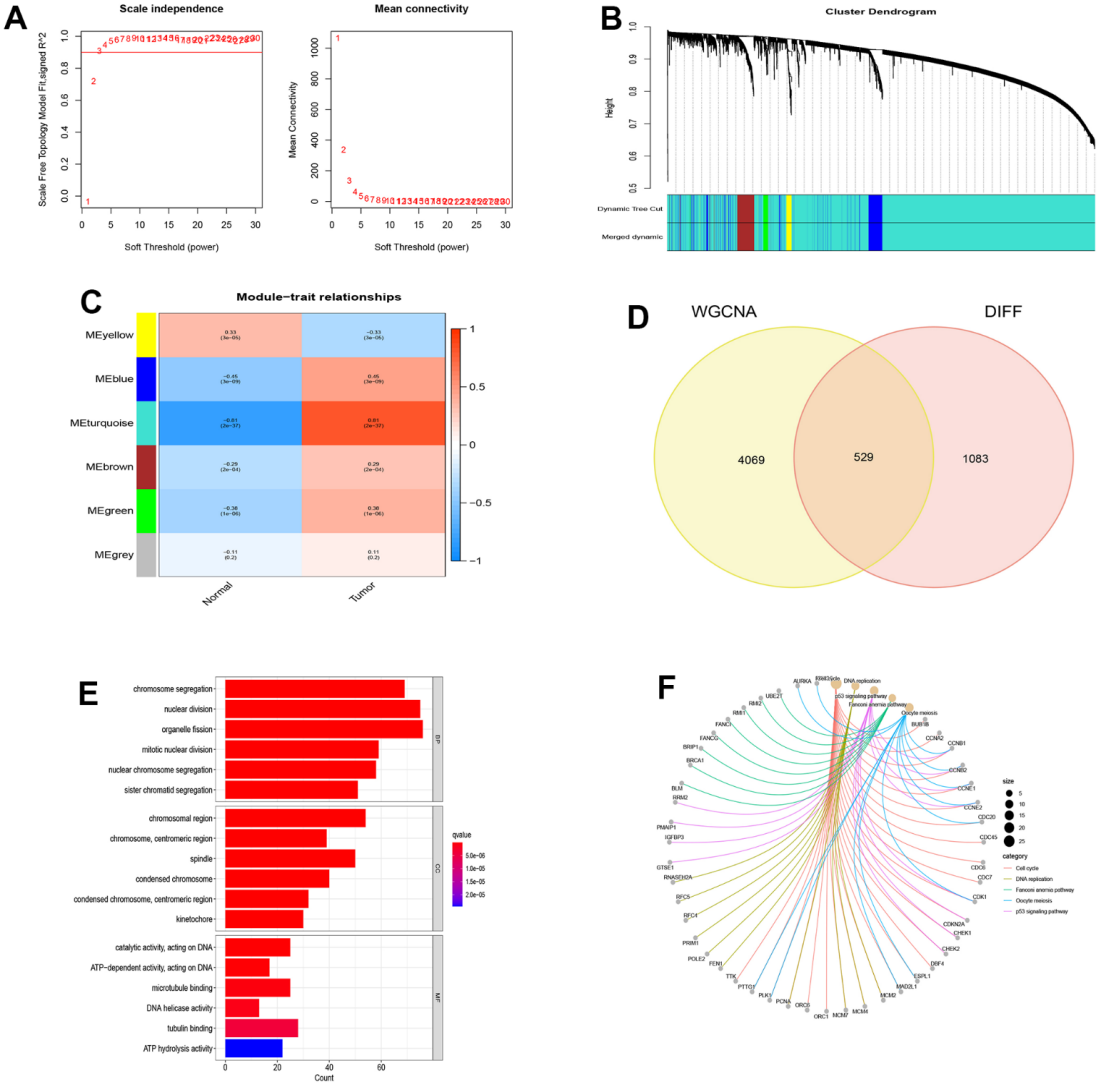
**WGCNA analysis and module identification.** (**A**) Scale-free exponent and average connectivity of different soft threshold powers (β); (**B**) Construction of gene co-expression modules; (**C**) Correlation analysis between different modules and tumor tissues; (**D**) Venn diagram of WGCNA and differentially expressed genes; (**E**) GO analysis of disease genes; (**F**) KEGG analysis of disease genes.

From the NSCLC data set (GSE19188), we screened out 1612 differentially expressed genes compared with normal tissues. Then, we selected a correlation coefficient of 0.86 corresponding to a soft threshold of 3 to construct a scale-free network for WGCNA analysis (Figure 2A, 2B). Through WGCNA analysis, all genes of NSCLC were constructed into 6 different gene modules, namely MEyellow, MEblue, MEturquoise, MEbrown, MEgreen and MEgrey, as shown in Figure 2C. According to the correlation analysis between the gene modules and Tumor shown in Figure 2C, These gene modules are related to the occurrence and development of NSCLC, among which MEturquoise has the most significant correlation with NSCLC and is considered as a key gene module. Based on the intersection of genes in this module and differentially expressed genes, 529 disease-causing genes were found. These pathogenic genes were mainly enriched in organelle fission (BP), chromosomal region (CC) and catalytic activity acting on DNA (MF) in functional enrichment analysis. At the same time, KEGG analysis showed that these pathogenic genes were closely related to cell cycle, DNA replication and P53 signaling pathway (Figure 2D–2F).

### PPI network of drug targets and disease genes

**Figure 3:**
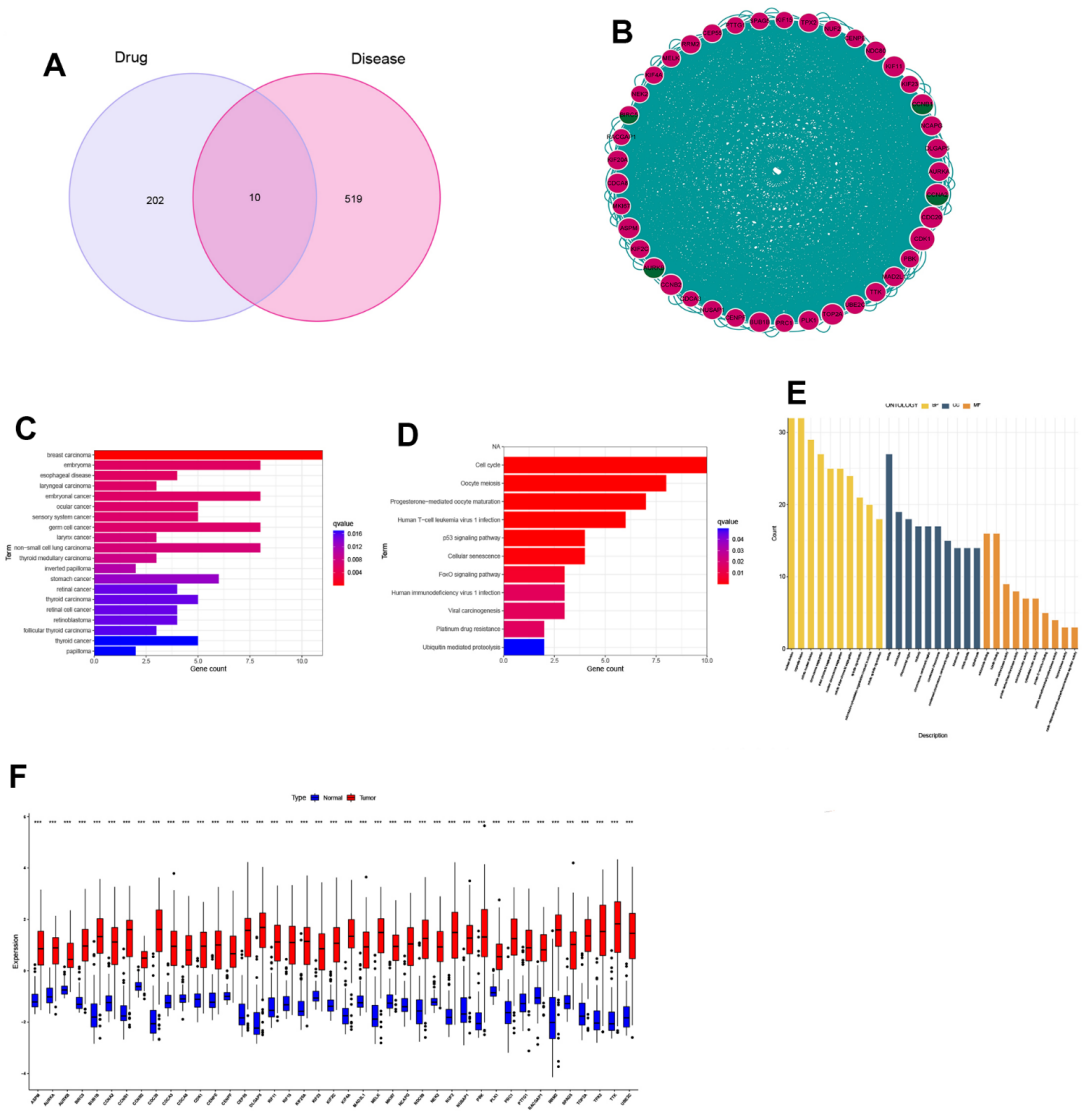
**Identification and expression analysis of hub genes.** (**A**) Venn diagram of drug targets and disease genes; (**B**) Protein-protein interaction (PPI) network of 41 hub genes; (**C**) DO analysis; (**D**) KEGG analysis; (**E**) GO analysis; (**F**) Gene expression between normal and tumor tissues.

Through the combination of drug targets and disease genes, 10 genes were found to be drug-disease genes (Figure 3A). The first PPI network was obtained based on the STRING database and Cytoscape. The subnetwork of 124 genes was obtained by the default setting of the MCODE plug-in in Cytoscape, using MCODE again and 41 hub genes were further screened out, including 4 drug-disease genes (Figure 3B). To explore the functional annotations of these hub genes, we performed DO, KEGG and GO enrichment analyses. DO analysis shows that these genes are still highly enriched for NSCLC (Figure 3C). The KEGG pathway enrichment analysis results are shown in Figure 3D. For the GO functional annotation of genes, the top 10 annotation results of BP, CC, and MF are shown in the Figure 3E. At the same time, the expression comparison between tumor tissue and normal tissue found that these hub genes were highly expressed in tumor tissue (Figure 3F). The results of ROC analysis showed that all 41 genes had good diagnostic performance for NSCLC (Supplementary Figures 1, 2).

### Analysis of tumor cell immune infiltration in HNSCC

**Figure 4:**
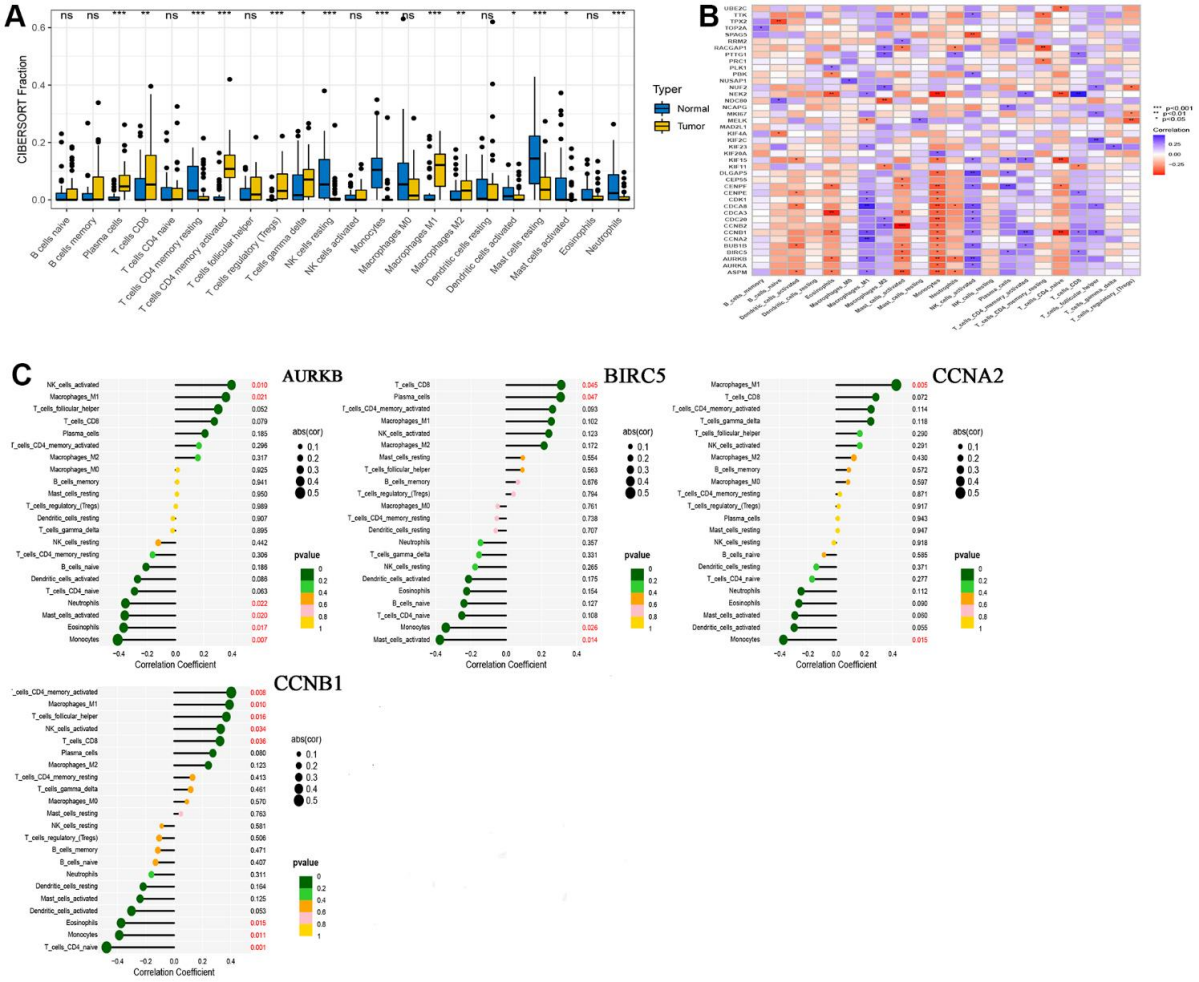
**CIBERSORT analysis results.** (**A**) Comparison of immune cell infiltration in normal and tumor tissues; (**B**) Correlation between 41 hub genes and immune cells; (**C**) A lollipop graph of the relationship between 4 drug-disease genes and different immune cells.

We performed immune infiltration analysis for normal and tumor groups using CIBERSORT algorithms, and the results are shown in Figure 4A. These calculation results allow us to intuitively understand that there are significantly more Plasma cells, various types of T cells, Macrophages M1 and Macrophages M2 in tumor tissues. The results of correlation analysis between 41 hub genes and immune cells are shown in Figure 4B. At the same time, we found that four drug-disease genes (CCNB1, CCNA2, BIRC5 and AURKB) were negatively correlated with monocytes, and had statistical significance, but showed different correlations with other types of immune cells (Figure 4C).

### Single cell sequencing and drug-disease gene analysis

**Figure 5:**
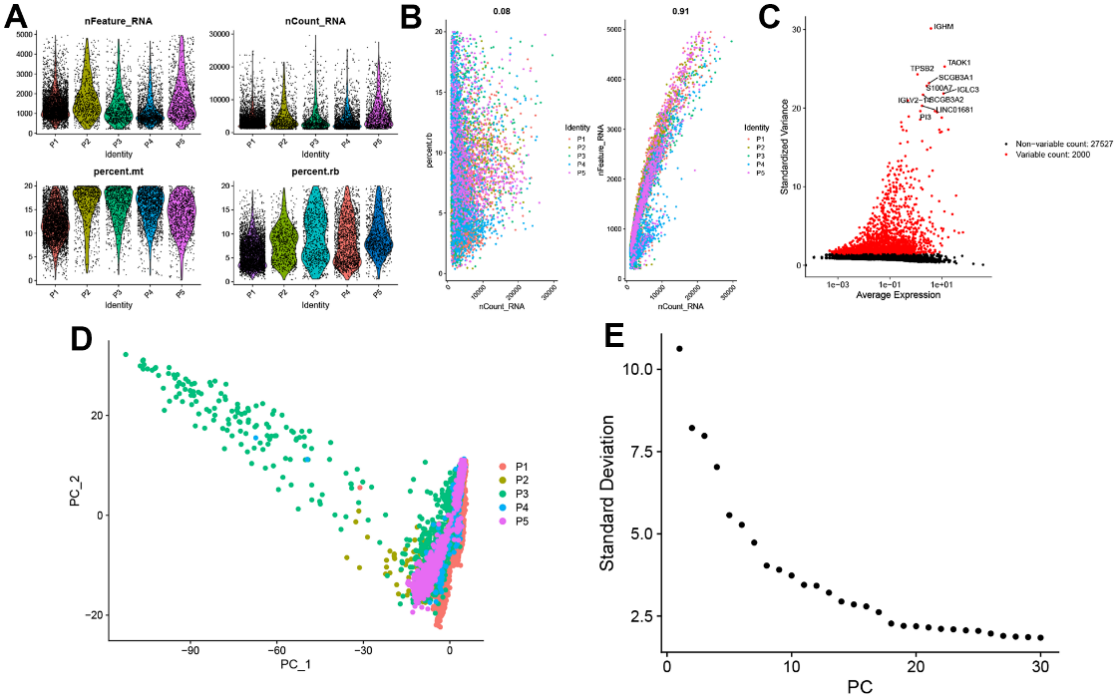
**Single-cell sequencing data quality control and dimensionality reduction.** (**A**) Relationship between mRNA/UMI/mitochondrial content/rRNA content of each sample after filtration. (**B**) The relationship between rRNA and UMI, and the relationship between mRNA and UMI; (**C**) The volcano map of the top 2000 hypervariable genes; (**D**) PCA dimensionality reduction sample distribution map; (**E**) PCA anchor point map.

**Figure 6:**
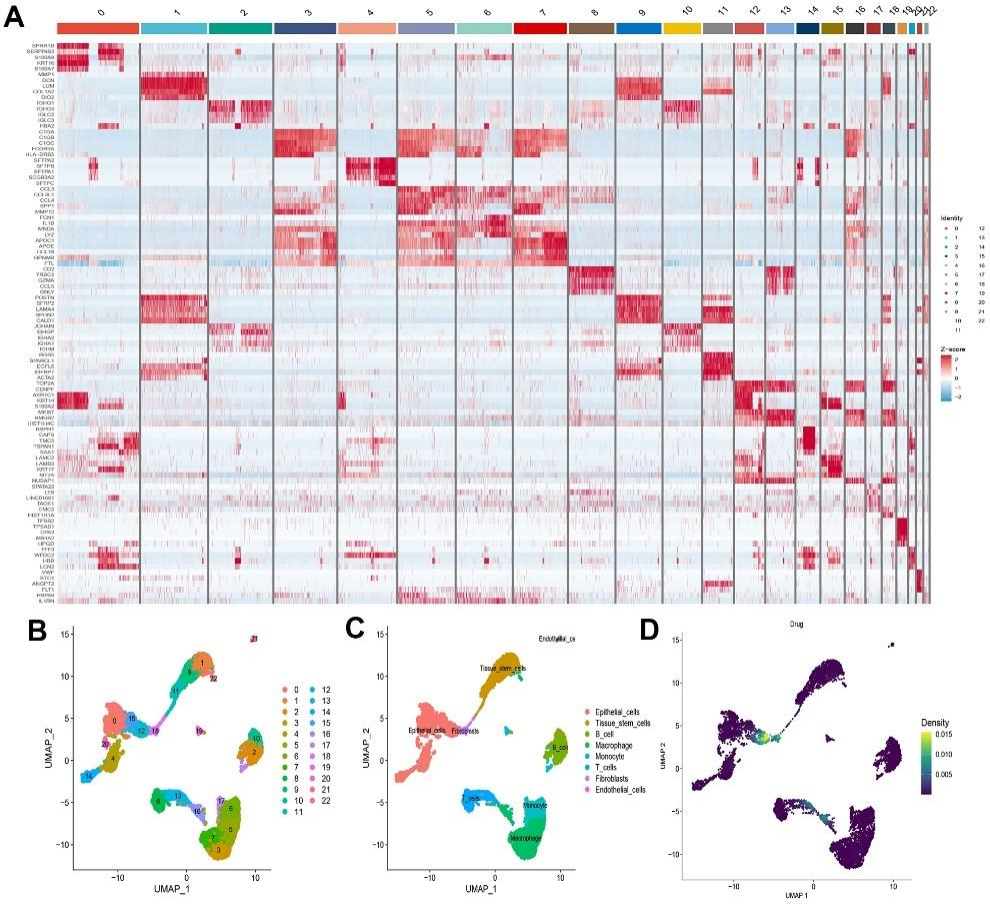
**Definition and analysis of cell clusters.** (**A**) Heatmap of differentially expressed genes in different cell clusters; (**B**) Uniform Manifold Approximation and Projection (UMAP) of 23 cell clusters; (**C**) The cell types were identified by marker genes; (**D**) Distribution of 4 drug-disease genes in different cells.

**Figure 7:**
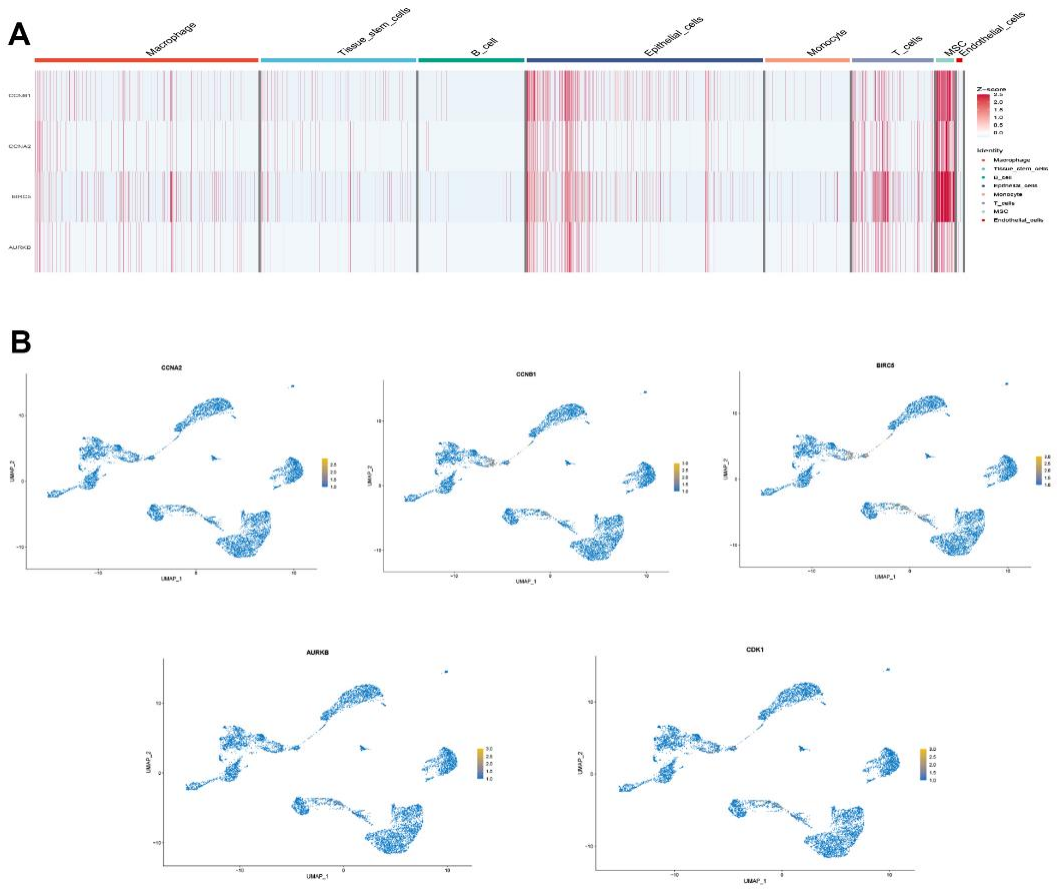
**Drug-disease genes and single cell sequencing.** (**A**) Heat map of expression of 4 genes among 8 types of cells; (**B**) Display of the distribution of each gene.

We selected the first five samples of the single-cell dataset (GSE148071), and visualized the QC metrics as a violin after following the quality control (QC) criteria of expressing more than 200 genes and less than 10,000 genes per cell, and mitochondrial content <20% (Figure 5A), while finding a positive correlation with total intracellular sequences (R = 0.91, Figure 5B), we also found a slight correlation between sequencing depth and rRNA (R = 0.08). A total of 2,000 highly variable features were identified from 27,527 features (Figure 5C). When performing PCA dimensionality reduction screening, we selected 2000 highly variable features as input, and finally selected 20 PCs with significant differences for further analysis (Figure 5D, 5E). Using the UMAP algorithm, we clustered the cells into 23 clusters and identified 15058 marker genes. In the heatmap, the top 10% of marker genes in each cluster are represented (Figure 6A). 23 clusters were annotated according to marker genes: cluster 2/10 is B cells, cluster 21 is Endothelial cells, cluster 0/4/12/14/15/20 is Epithelial cells, cluster 18 is Fibroblasts, cluster 3/5/7/16/22 is Macrophage, cluster 6/17/19 is Monocyte, cluster 8/13 is T cells, cluster 1/9/11 is Tissue stem cells (Figure 6B, 6C). The up-regulated differentially expressed genes were retained by the FindAllMarkers function and combined with the four drug-disease genes in the hub genes to analyze the distribution of these genes in cell subtypes (Figure 6D). According to the data in Figure 7A, 7B, it can be known that these four genes are highly expressed in epithelial cells, macrophages, T cells and fibroblasts.

### SMR analysis reveals potential key gene in NSCLC development

**Figure 8:**
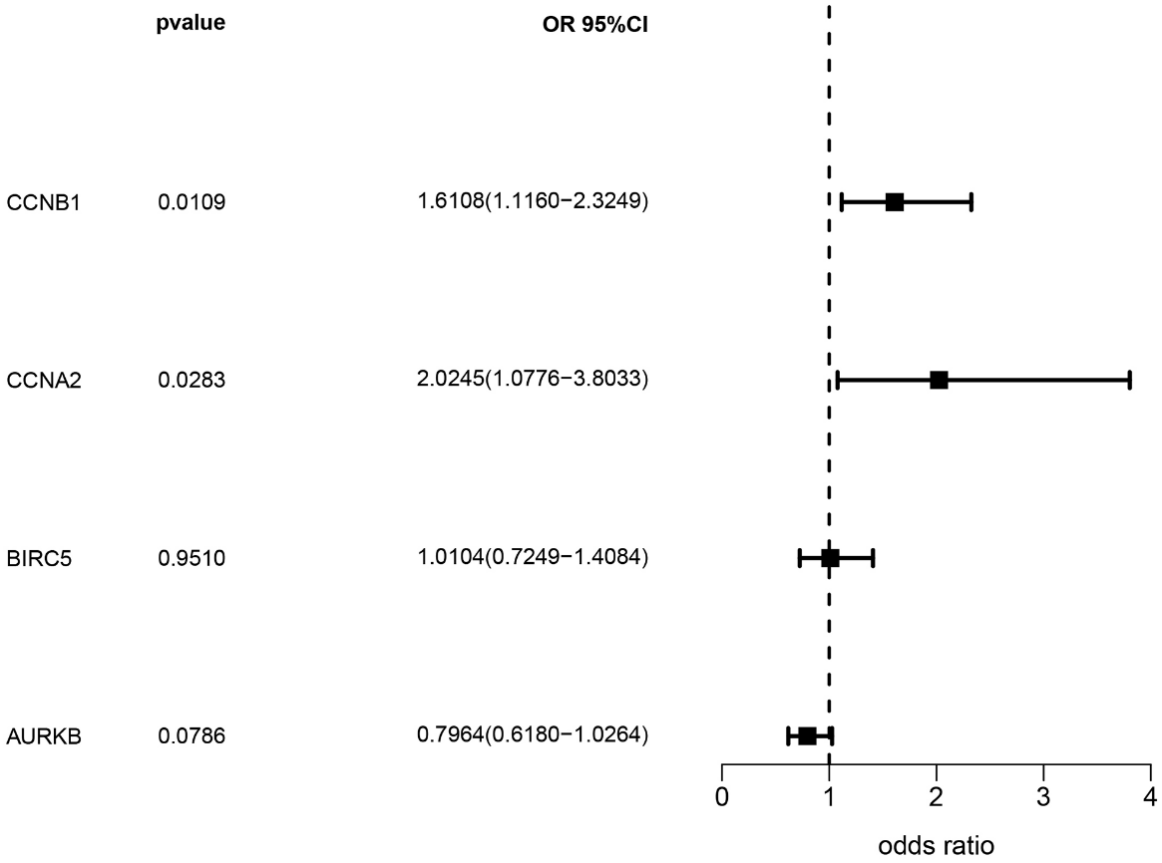
Analysis results of SMR.

Based on the eQTLGen dataset, a total of 79, 87, 106, and 174 cis-eQTLs were identified for the CCNB1, CCNA2, BIRC5, and AURKB genes associated with the drug-disease relationship. The most significant cis-eQTLs were selected as genetic tools for targeting these genes. In Figure 8, the results of the SMR analysis revealed a significant increase in gene expression of CCNA2 and CCNB1 in blood samples associated with an increased risk of NSCLC occurrence. Specifically, CCNA2 showed an odds ratio (OR) of 2.024 (*95%* confidence interval [CI]: *1.078–3.803; P = 0.028*), suggesting its potential importance in the development of NSCLC and warranting further investigation. However, although BIRC5 and AURKB showed a trend towards association with NSCLC occurrence, the corresponding P-values did not reach statistical significance. Table 1 presents the representative eQTLs for each gene and the results of the heterogeneity test (HEIDI), which indicate no significant heterogeneity among the selected eQTLs (*P ≥ 0.01*).

**Table 1:** Representative eQTL for SMR genes and HEIDI tests.

**Gene**	**Chromosome location**	**Top eQTL**	**F-statistic**	**HEIDI (P-value)**	**HEIDI (Number of eQTLs)**
CCNB1	5	rs352626	256.70482	3.37E-01	16
CCNA2	4	rs4833235	87.343871	3.66E-02	4
BIRC5	17	rs11077350	266.91761	4.13E-01	4
AURKB	17	rs12938531	246.57431	3.45E-01	20

### Tetrandrine reduced the viability, proliferation as well as induced the cell death in NSCLC cells

**Figure 9:**
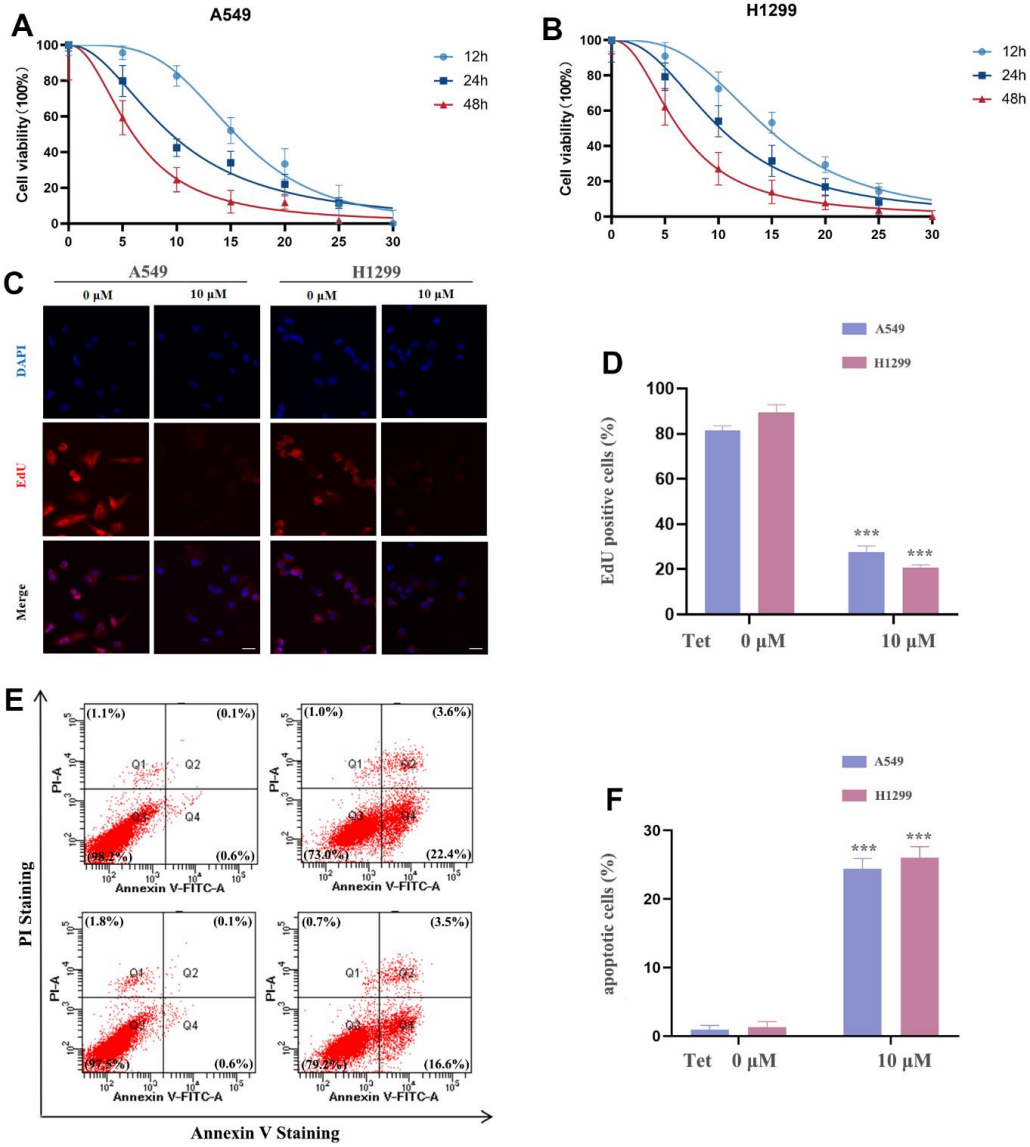
**The effect of tetrandrine treatment on NSCLC cells.** (**A**, **B**) NSCLC cells were subjected to several concentrations of tetrandrine treatment and the cell viability was measured by CCK-8 assay; (**C**, **D**) Cell proliferation of NSCLC cells were conducted by 5-ethynyl-2′-deoxyuridine (EdU) incorporation assay; (**E**, **F**) NSCLC cells were subjected to tetrandrine treatment (10 μm, 24 h) and followed by Annexin V-FITC/PI assay. *P < 0.05, **P < 0.01, ***P < 0.001 versus control.

In order to explore the anticancer properties of tetrandrine on NSCLC, different doses of tetrandrine (0-30 μM) were treated in the NSCLC cells. Interestingly, we found that tetrandrine had the potentiality to restrain the viability of NSCLC cells (Figure 9A, 9B). And the IC50s in A549 and H1299 cells were 9.50 μM and 10.18 μM, respectively. Therefore, 10 μM treatment of tetrandrine was selected for the next experiment. Next, we wonder whether tetrandrine treatment could suppress NSCLC cell proliferation. As expected, tetrandrine treatment caused a remarkable reduction in the percentage of EdU+ cells compared with the ctrl-group, reveling that tetrandrine significantly disrupted NSCLC growth (Figure 9C, 9D). Consequently, we utilized the Annexin V-FITC/PI assay to further clarify the potential effect of tetrandrine, and the experiment demonstrated that tetrandrine treatment significantly increased the amounts of dead cells (Figure 9E, 9F). Collectively, these results highlighted the anticancer effect of tetrandrine.

### Molecular docking of tetrandrine with CCNA2 and CCNB1

**Figure 10:**
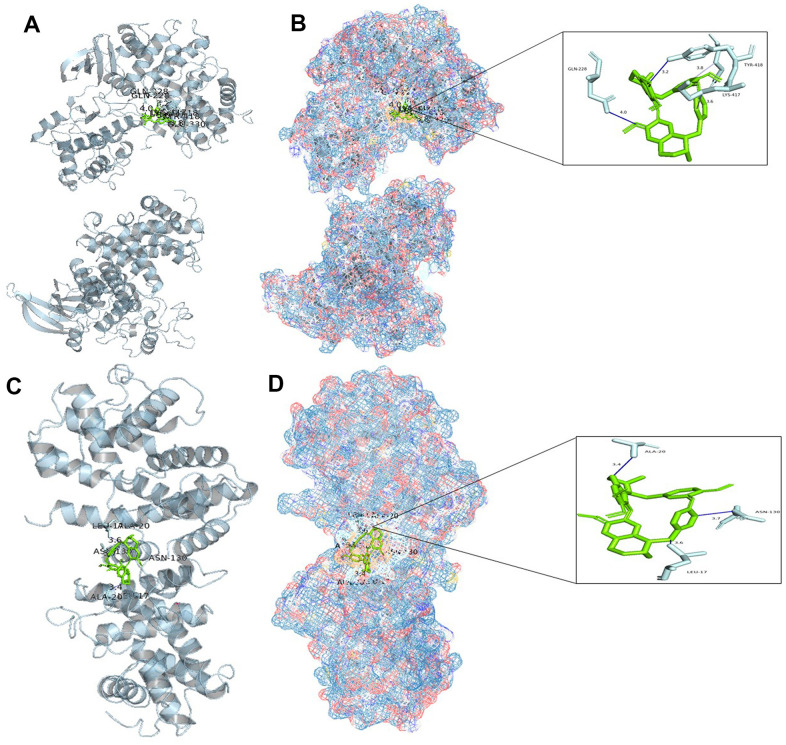
**Molecular docking analysis.** (**A**) Binding mode of CCNA2 protein and tetrandrine; (**B**) Three-dimensional (3D) interaction map of CCNA2 protein and tetrandrine; (**C**) Binding mode of CCNB1 protein and tetrandrine; (**D**) Three-dimensional (3D) interaction map of CCNB1 protein and tetrandrine.

Based on the above analysis, CCNB1, CCNA2, BIRC5, and AURKB are considered to be important genes for tetrandrine in NSCLC treatment. Single-cell sequencing results show that CCNA2 and CCNB1 are distributed in epithelial cells, and SMR shows that CCNA2 and CCNB1 are closely related to the occurrence of NSCLC. So we decided to carry out molecular docking on them. We identified 20 molecular docking patterns of tetrandrine with CCNA2 and CCNB1 (Table 2). Through the Protein-Ligand Interaction Profiler online platform, the analysis indicates that CCNA2 exhibits hydrogen bonding and hydrophobic interactions with tetrandrine (Figure 10A, 10B). In contrast, CCNB1 demonstrates hydrophobic interactions with tetrandrine (Figure 10C, 10D), consolidating the possibility of tetrandrine’s modulation through these specified genes. All procedures of this study can be reviewed in Supplementary Figure 3.

**Table 2:** CCNA2 and CCNB1 molecular docking results.

**Mode**	**Affinity(kcal/mol) (CCNA2)**	**Affinity(kcal/mol) (CCNB1)**
1	-8.2	-7.5
2	-7.9	-7.4
3	-7.7	-7
4	-7.6	-6.8
5	-7.5	-6.8
6	-7.2	-6.7
7	-7.1	-6.6
8	-7.1	-6.6
9	-7.1	-6.6
10	-7.1	-6.5
11	-7.0	-6.5
12	-6.8	-6.5
13	-6.8	-6.5
14	-6.7	-6.3
15	-6.6	-6.3
16	-6.5	-6.2
17	-6.5	-6.2
18	-6.4	-6.2
19	-6.4	-6
20	-6.3	-5.9

## DISCUSSION

Although the current treatment methods for non-small cell lung cancer include surgery, radiotherapy, chemotherapy, targeted therapy, and immunotherapy, the treatment of NSCLC patients remains a serious challenge due to emerging toxic side effects and drug resistance [31–34]. Traditional Chinese medicine has a long history of treating lung cancer, and in-depth exploration of the role of traditional Chinese medicine in cancer treatment may provide us with a new breakthrough.

In a previous study, a series of novel tetrandrine derivatives were successfully synthesized via the Suzuki-Miyaura reaction and their cytotoxicity against human non-small cell lung cancer (NSCLC) A549 cells was evaluated. The results showed that compounds Y5, Y6, Y9 and Y11 had the most significant cytotoxic effects, with IC50 values ranging from 3.87 to 4.66 mM. This discovery will help in the design of more effective chemotherapy drugs for lung cancer in the future [35]. Some scholars found that inhibition of mitochondrial ATP production in lung adenocarcinoma A549 cells can enhance the cytotoxicity of tetrandrine [36]. Studies have pointed out that tetrandrine may increase the sensitivity of PC14 cells to gefitinib through lysosome inhibition [37].

We observed that tetrandrine could inhibit the viability and proliferation of NSCLC cells and induce cell death. However, the relationship between drugs and diseases is very complex. We aim to explore their relationship in various ways. 213 drug target genes were got from four drug target databases, and DO analysis confirmed that these drug targets mainly play a role in various cancers including NSCLC. KEGG analysis confirmed that these genes were enriched in EGFR tyrosine kinase inhibitor resistance [38], PI3K-Akt signaling pathway [39], PD-L1 expression and PD-1 checkpoint pathway [40] and other pathways. GO analysis also confirmed that drug genes were enriched in peptide-serine modification, synaptic membrane and protein serine/threonine kinase activity. Many studies have confirmed that the above enrichment results are closely related to NSCLC [41, 42]. Then we adopted the method of WGCNA interacting with differentially expressed genes. Finally, we obtained 529 disease genes.

Using the interactions between drug targets and disease genes, we identified 10 drug-disease genes and constructed a PPI network covering all drug and disease genes. Through two rounds of MCODE screening, we screened out a core gene network containing four drug-disease genes. We performed DO/GO/KEGG functional enrichment analysis on these core genes and found that these genes were strongly associated with the progression of NSCLC. In particular, KEGG analysis results showed that these core genes were enriched in pathways such as platinum resistance [43], Cellular senescence [44] and P53 signaling pathway [45], This also suggests that the hub gene may affect NSCLC through those ways.

After immune infiltration analysis, we found that the number of various types of T cells and macrophages increased in tumor tissues. On the one hand, it shows that the antigens expressed by tumor cells can be recognized by the immune system, thereby activating T cells and macrophages. On the other hand, tumor cells secrete a variety of growth factors and cytokines, such as interleukin 6 (IL-6), tumor necrosis factor-α (TNF-α) and interferon-γ (IFN-γ), which may also lead to such results [46, 47]. At the same time, we found that four drug-disease genes (CCNB1, CCNA2, BIRC5, and AURKB) were highly expressed in tumors, with high diagnostic accuracy, and were positively correlated with plasma cells, T cells, and macrophages; while negatively correlated with monocytes. Studies have shown that T cells and B cells play an important role in tumor immunity [48, 49]. If these drug-disease genes can be used as new biomarkers, it may provide new ideas for current immunotherapy.

Single-cell sequencing results showed that multiple cell types still exist in NSCLC at the cellular level. In addition, four genes showed higher expression in epithelial and endothelial cells compared to other cell types. Combined with previous bioinformatics data, suggest that they may be involved in specific biological processes in epithelial and endothelial cells, such as cell proliferation, metastasis and angiogenesis, which play a crucial role in the development and progression of NSCLC. To overcome the limitations of traditional observational studies, the SMR approach was used in this study, utilizing genetic variants associated with the expression of four genes as instrumental variables. The analysis showed that increased expression of CCNA2 and CCNB1 was associated with an increased risk of NSCLC formation. Research has revealed a significant upregulation of CCNA2 mRNA and lncRNA DNAH17 antisense RNA 1 (DNAH17-AS1) in NSCLC specimens and cell lines, accompanied by a notable decrease in miR-877-5p expression. Elevated DNAH17-AS1 levels correlate with TNM staging, distant metastasis, and reduced overall survival. Functional analysis indicates that silencing DNAH17-AS1 inhibits proliferation, migration, and invasion of H1299 and 95D cells while promoting apoptosis. Further mechanistic investigations suggest that DNAH17-AS1 may function as a miR-877-5p sponge to upregulate CCNA2, thereby exerting its oncogenic role [50]. Simultaneously, research has also shown that Danshensu IIA can inhibit the progression of lung adenocarcinoma by inducing apoptosis and cell cycle arrest. These effects are achieved through the regulation of the CCNA2-CDK2 complex and the AURKA/PLK1 pathway [51]. Previous studies have indicated that MEOX1 exhibits lower expression in lung cancer tissues compared to normal adjacent tissues. MEOX1 shows a positive correlation with the overall survival of lung cancer patients, particularly those with lung adenocarcinoma. *In vitro* and *in vivo* functional experiments have demonstrated that stable overexpression of MEOX1 significantly inhibits the proliferative capacity of NSCLC cells, induces cell cycle arrest in the G2 phase, and enhances apoptotic capabilities. Furthermore, researchers have found a negative correlation between MEOX1 and CCNB1 mRNA expression in various lung cancer tissues. Further investigations have revealed that MEOX1 suppresses the progression of lung cancer cells by inhibiting the cell cycle checkpoint gene CCNB1 [52]. Bao et al. [53] discovered that overexpression of CCNB1 promotes the progression of lung cancer cells, and miR-139-5p may act as a negative regulator of CCNB1, thereby inhibiting cell proliferation, migration, invasion, and cell cycle progression. Our molecular docking studies of CCNA2 and CCNB1 with tetrandrine have revealed interaction sites, highlighting the significant potential of tetrandrine in the treatment of NSCLC. This presents a crucial opportunity for repurposing as compared to the development of new drugs.

## CONCLUSIONS

In this study, we systematically investigated the relationship between tetrandrine and non-small cell lung cancer (NSCLC), confirming the relevance and importance of drug-disease genes in tumor immunity, cell subtypes, and disease progression. It is also well illustrated that the repurposing of tetrandrine could potentially contribute to the treatment of NSCLC.

## Supplementary Material

Supplementary Figures
