# Supplementary material for: New perspectives on the potential of tetrandrine in the treatment of non-small cell lung cancer: bioinformatics, Mendelian randomization study and experimental investigation
Source: Aging (Albany NY). 2024 Jan 4;16(1):518–37. doi: 10.18632/aging.205384 (PMC10817384; doi:10.18632/aging.205384)
Supplement: Supplementary Figures [file aging-16-205384-s001.pdf]

SUPPLEMENTARY FIGURES

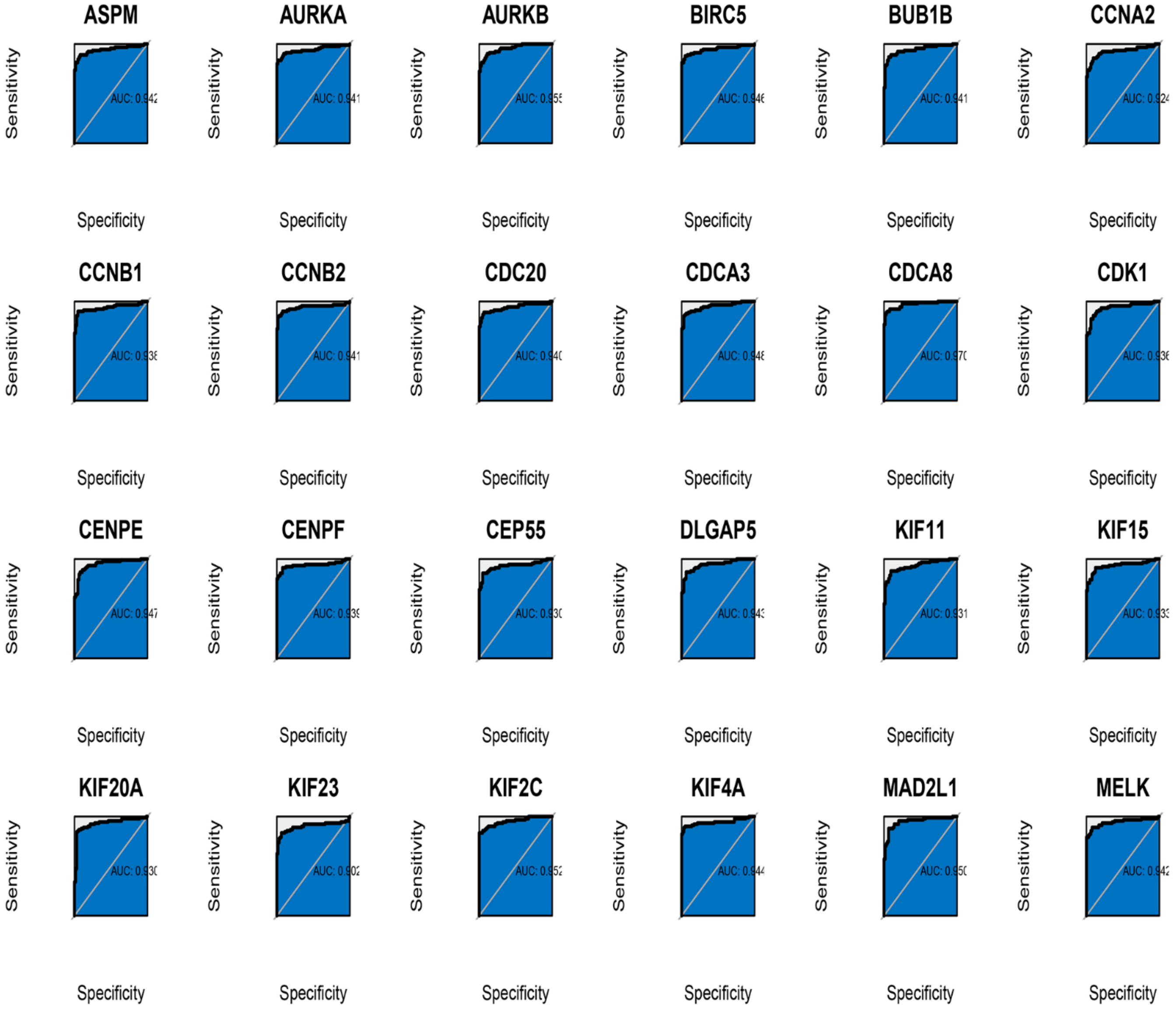

Supplementary Figure 1. ROC analysis of the first 24 genes.

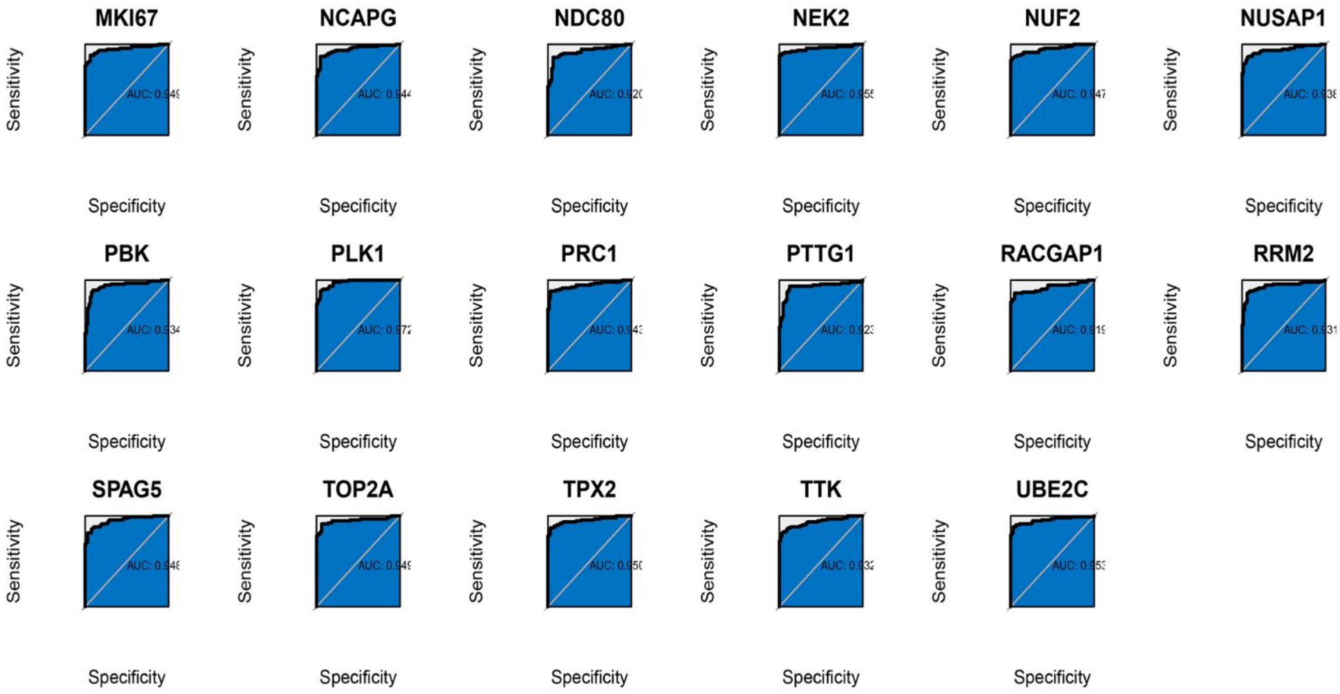

Supplementary Figure 2. ROC analysis of the subsequent 17 genes.

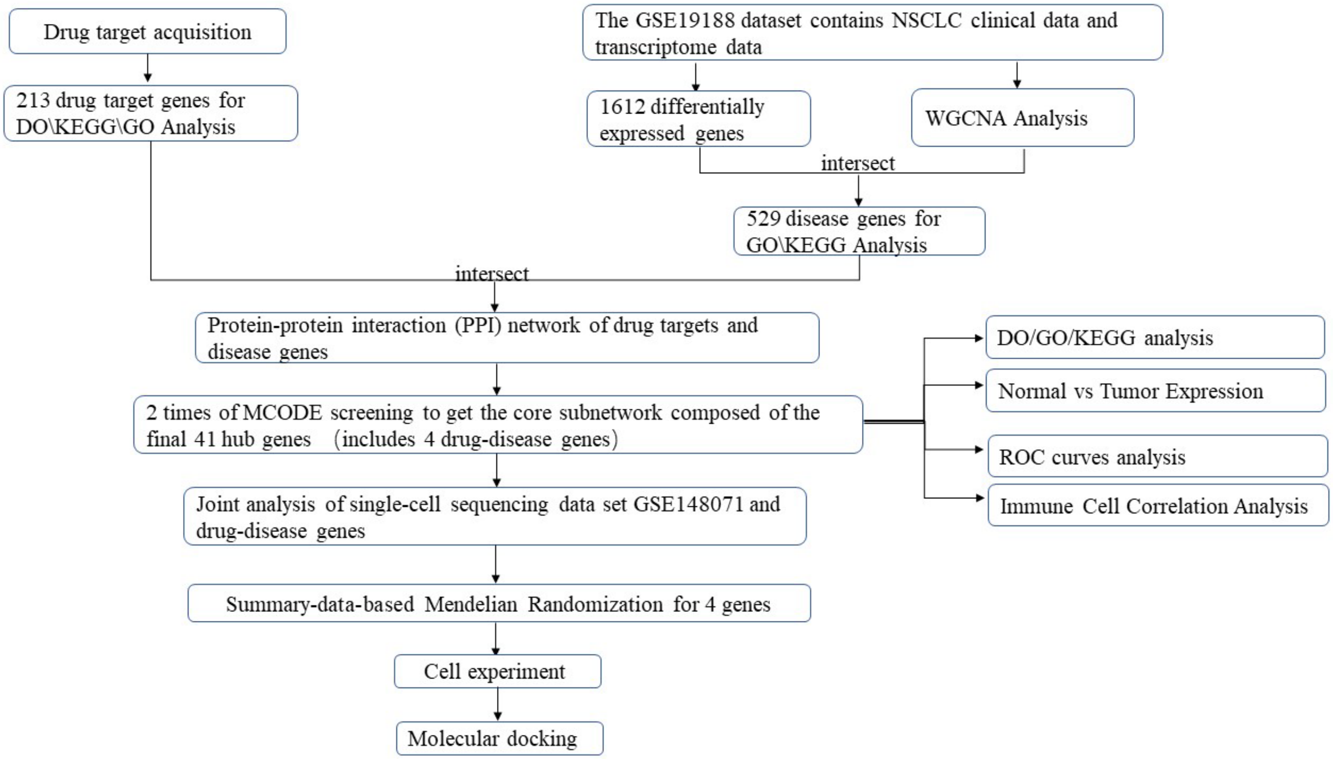

Supplementary Figure 3. Research flowchart.
